# Advances and Challenges in Tracking Interactions Between Plants and Metal-Based Nanoparticles

**DOI:** 10.3390/nano14231939

**Published:** 2024-12-03

**Authors:** Kena Zhang, Qingmeng Liu, Yukun Wang, Xigui Liu, Xiaoxia Zhou, Bing Yan

**Affiliations:** 1 School of Environmental Science and Engineering, Shandong University, Qingdao 266701, China; 2 Institute of Environmental Research at the Greater Bay Area, Key Laboratory for Water Quality and Conservation of the Pearl River Delta, Ministry of Education, Guangzhou University, Guangzhou 510006, China

**Keywords:** metal-based nanoparticles, plant uptake, extraction, mass spectrometry, biotransformation

## Abstract

Metal-based nanoparticles (MNPs) are increasingly prevalent in the environment due to both natural processes and human activities, leading to direct interactions with plants through soil, water, and air exposure that can have beneficial and detrimental effects on plant growth and health. Understanding the uptake, translocation, and transformation of MNPs in plants is crucial for assessing environmental risks and leveraging nanotechnology in agriculture. However, accurate analysis of MNPs in plant tissues poses significant challenges due to complex plant matrices and the dynamic nature of nanoparticles. This short review summarizes recent advances in analytical methods for determining MNP–plant interactions, focusing on pre-processing and quantitative nanoparticle analysis. It highlights the importance of selecting appropriate extraction and analytical techniques to preserve nanoparticle integrity and accurate quantification. Additionally, recent advances in mass spectrometry, microscopy, and other spectroscopic techniques that improve the characterization of MNPs within plant systems are discussed. Future perspectives highlight the need to develop real-time in situ monitoring techniques and sensitive tools for characterizing nanoparticle biotransformation.

## 1. Introduction

Plants inevitably come into contact with nanoparticles (NPs), which are widely present in the environment from both natural and anthropogenic sources [1]. The production of nanotechnology products has surged in the past few decades, resulting in 11,172 commercial products available on the market [2]. Due to their excellent optical, electromagnetic, and mechanical properties, metal-based NPs (MNPs) have become the most widely used nanomaterials, with diverse applications in medicine, catalysis, environmental remediation, antibacterial applications, and agricultural production [3,4,5,6,7]. Engineered MNPs can be intentionally or unintentionally released into the environment, such as through wastewater and sludge discharge or as nano-fertilizers applied to soil [8,9]. Considerable amounts of natural NPs are also formed in the environment each year [10], including MNPs and their oxides, hydroxides, and sulfides derived from wind erosion, weathering, volcanic activity, cosmic dust, biomineralization, and biomass combustion [1].

MNPs exhibit relatively high bioavailability, specifically those smaller than 20 nm. Compared to bulk materials, they can penetrate physiological barriers of plants such as the cuticle layer, stomatal pores, cell walls, cytomembranes, and plasmodesmata channels [11,12]. Moreover, when compared to metal ions or chelated forms, MNPs have a greater tendency to adhere to and remain on leaves and gradually absorbed [13].

The exposure of plants to MNPs can have positive or negative effects on their growth, resistance, and overall health [14,15,16]. The biological behavior of MNPs within plants—specifically uptake, translocation, and transformation—is a critical factor contributing to these effects. On the one hand, the design of MNP-based agricultural products should consider this. For instance, a stomata-targeted nano-pesticide based on gold nanoparticles (AuNPs) coated with LM6-M, an antibody with affinity for stomata, was used to kill pathogens that penetrate open stomata and spread to other parts of the plant [17]. On the other hand, the potential risks associated with MNP exposure are also a concern. Both foliar and root uptake of MNPs can interfere with a plant’s physiological health [16,18,19,20]. For example, foliar exposure to ceria oxide nanoparticles (CeO_2_ NPs) can disrupt photosynthesis, perturb nutrient acquisition, and reduce fruit firmness in cucumbers [21]. After root exposure, ytterbium oxide nanoparticles (Yb_2_O_3_ NPs) can inhibit root elongation and decrease the biomass of cucumber plants [22]. MNPs can also be translocated through the phloem or xylem and ultimately accumulate in edible plant parts, potentially resulting in trophic transfer and toxicity to higher trophic organisms [23,24,25,26]. Several studies have demonstrated that MNP transfer between lettuce and terrestrial snails, lettuce and hornworms, and algae–brine shrimp–guppy fish can cause oxidative damage to targeted organs, inhibit the individual activity of animals, reduce feces excretion, decrease embryo and larva numbers, and even lead to death [27,28].

The fate of MNPs in plants is also influenced by their biotransformation, as they are highly dynamic and unstable. Changes can occur in size, morphology, surface chemistry, and speciation, which are classified into three forms: chemical transformation, physical transformation, and biological transformation [29,30]. Zinc oxide nanoparticles (ZnO NPs, 9 nm) quickly entered the roots of maize and were deposited in vacuoles [31]. The acidic microenvironment of vacuoles dissolved ZnO NPs into Zn^2+^ ions, which not only allowed zinc to reach the stems and shoots but also induced toxicity, affecting plant growth, zinc uptake, dry biomass production, and root morphology.

To gain detailed insights into the interactions between MNPs and plants, it is necessary to develop suitable methods for determining the content, location, and biological behaviors of MNPs. However, traditional analytical tools used in nanotechnology and plant science face two significant challenges: the complex and polydisperse matrices of plant tissues, and the heterogeneity of MNPs undergoing various unclear transformations during environmental processes [25]. To overcome matrix interference, MNPs can be extracted from plant tissues before analysis, but preserving their properties during extraction is crucial [32]. Alternatively, in situ analysis with high selectivity and sensitivity is a viable choice [33,34]. To effectively analyze the complex MNPs in plants, the highest possible spatial and chemical resolution is necessary, and multiple analytical techniques are usually required in most cases.

This review focuses on the most recent advances in analytical methods for determining MNP uptake by plants, emphasizing the importance of extraction and other pre-processing techniques. The applications of various analytical methods—including mass spectrometry (MS), microscopy, and spectroscopy—in studying MNP interactions with plants will be discussed and compared with specific examples. Additionally, the main challenges for future research are analyzed.

## 2. Extraction of MNPs from Plant Tissues

Quantitative analysis of MNPs in plants requires effective extraction of NPs from complex biological matrices while preserving their physicochemical properties. The choice of extraction method significantly impacts the accuracy of quantification and characterization.

### 2.1. Acid Digestion

Acid digestion is commonly used for releasing and collecting metals from plant tissues, particularly for total metal quantification using inductively coupled plasma optical emission spectroscopy (ICP-OES) or mass spectrometry (ICP-MS) [35]. Concentrated acids such as nitric acid (HNO₃), hydrochloric acid (HCl), hydrofluoric acid (HF), or their mixtures are employed based on the specific MNPs, plant materials, and instrument parameters [21,36]. Acid digestion [37] was used to release molybdenum disulfide nanoparticles (MoS_2_ NPs) from soybean plant roots, shoots, and nodules. By using a mixture of HNO₃ and H_2_O_2_, the study authors were able to dissolve the plant tissues and analyze the total molybdenum content using ICP-MS. The study highlighted that while acid digestion is effective for total metal analysis, it may not preserve the NP form of labile MNPs like MoS_2_ NPs.

### 2.2. Enzymatic Extraction

Enzymatic extraction utilizes enzymes such as cellulase, hemicellulase, and pectinase to digest plant cell walls gently, effectively releasing MNPs in their particulate form [38,39,40]. A multi-component enzyme mixture, Macerozyme R-10, is commonly used for sample digestion. For example, AuNP particle recovery of 96% was achieved from lettuce leaves, with size distributions matching those of the original NPs, as determined using single-particle ICP-MS (spICP-MS) [38]. However, the acidic environment required for enzymatic activity may lead to the partial dissolution of some MNPs. For example, Macerozyme R-10 extraction from lettuce leaves has been shown to significantly reduce the average diameter of CuO NPs from 67 nm to 47 nm, as determined using spICP-MS [41].

### 2.3. Organic Solvent-Based Extraction

Organic solvents, such as methanol (MeOH), have been introduced as alternatives to enzymatic extraction to avoid issues related to pH and incomplete digestion [32,41,42]. MeOH can disrupt plant tissues and extract MNPs with minimal changes in size distribution, as shown by a previous analysis of MeOH-extracted copper-based nanoparticles (CuO NPs and Cu(OH)_2_ NPs) from lettuce leaves using spICP-MS [42].

### 2.4. Challenges, Method Comparison, and Future Directions

The current extraction methods and their typical applications are summarized in Table 1. The main challenges in extracting MNPs from plant tissues include preserving NP integrity, preventing dissolution or aggregation, and achieving complete recovery from complex matrices.

Acid digestion effectively releases total metals but often alters or dissolves NPs, making it unsuitable for studies requiring intact NPs [43]. Enzymatic extraction is gentle and preserves NPs’ properties better than acid digestion. However, it may not fully digest lignin-rich tissues and can cause dissolution of sensitive MNPs at low pH [41,44]. Organic solvent-based extraction offers an alternative that avoids low pH conditions and can preserve NP integrity, but its efficiency may vary depending on tissue characteristics and NP properties [41].

Developing effective standardized extraction protocols across a wide range of plant tissues and NP types remains a significant challenge. Future research should optimize extraction conditions, such as enzyme mixtures, solvent compositions, and mechanical disruption techniques, to improve recovery rates and preserve NPs’ properties. Additionally, understanding the interactions between NPs and plant matrix components will aid in developing more effective extraction methods. With effective extraction and pre-processing, quantitative analytical methods are needed to investigate how MNPs interact with plants. 

## 3. Mass Spectrometry-Based Analysis

MS is a powerful analytical technique for quantifying and characterizing MNPs due to its sensitivity, specificity, and ability to provide information on the mass-to-charge ratios of atoms and molecules [45]. Various MS-based techniques have been developed to analyze MNPs in plant tissues.

### 3.1. Inductively Coupled Plasma Mass Spectrometry (ICP-MS)

ICP-MS combines an inductively coupled plasma source with MS, enabling multi-elemental detection with high sensitivity (detection limits reaching pg/g levels) [46].

#### 3.1.1. Single Particle ICP-MS (spICP-MS)

spICP-MS allows for the detection and characterization of individual NPs in a suspension by introducing a dilute sample into the ICP-MS, enabling particles to be detected one at a time [47,48,49]. A previous study used spICP-MS to quantify the particle size distribution and concentration of CeO_2_ NPs extracted from radish plants, revealing differences in NP uptake and translocation within the plant [50].

#### 3.1.2. Laser Ablation ICP-MS (LA-ICP-MS)

**Figure 1:**
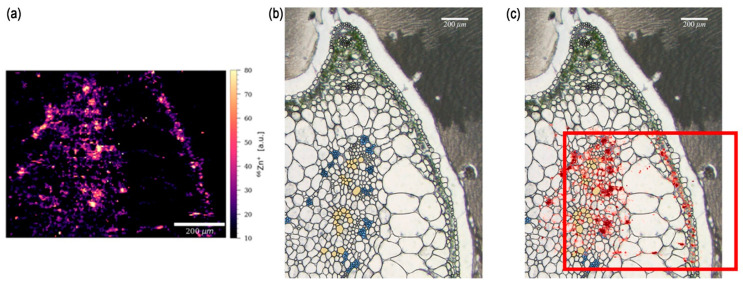
LA-ICP-MS imaging for the distribution of MNPs in tomato stems. (**a**) LA-ICP-MS image of Zn near the phloem and xylem tissues of the petiole connected to a leaf dosed with ZnO@MSN. (**b**) The area of the LA-ICP-MS scan with the phloem (blue) and xylem (yellow) regions. (**c**) Overlay of the LA-ICP-MS Zn signal (in red) with the microscopy image (**b**), the area of the sample that LA-ICP-MS analyzed in (**a**) is marked in the red square frame of (**c**). Reprinted with permission from [51]. Copyright 2023 American Chemical Society.

LA-ICP-MS enables spatially resolved analysis of elements within solid samples by ablating material from the sample surface and analyzing the emitted ions [51,52,53]. LA-ICP-MS was used to map ZnO NPs in tomato leaves and rice roots [51,54]. Using Zn as a tracer element, the study authors visualized NP distribution and translocation pathways within the plant tissue, demonstrating the utility of LA-ICP-MS in the in situ imaging of MNPs (Figure 1) [51].

#### 3.1.3. Other ICP-MS-Based Techniques

The hyphenation of ICP-MS with separation techniques, such as chromatography, allows for the differentiation of ionic and particulate metal forms. HPLC-ICP-MS has developed to separate AuNPs from ionic gold in algal cell lysates [55]. This approach enabled the study authors to evaluate changes in NP size and speciation after uptake by the algae, highlighting the potential of chromatographic separation coupled with ICP-MS in studying NP transformations.

### 3.2. Secondary Ion Mass Spectrometry (SIMS)

**Figure 2:**
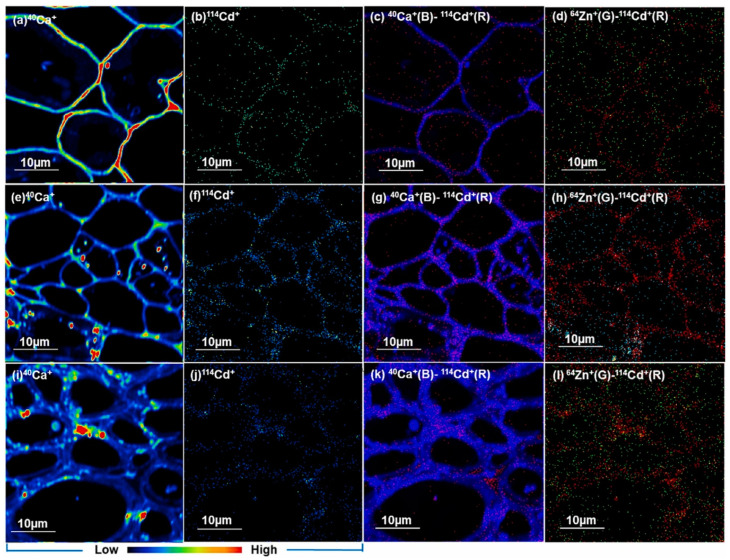
NanoSIMS imaging of the distribution of MNPs in chili plants. NanoSIMS elemental maps (10 µm × 10 µm) of chili (**a**,**b**) leaf, (**e**,**f**) stem, and (**i**,**j**) root tissues after foliar CdS NP exposure obtained using O^−^ beam polarities to map ^40^Ca^+^ and ^114^Cd^+^. (**c**,**d**,**g**,**h**,**k**,**l**) show the composite (multi) elemental maps of the chili leaf, stem, and root, respectively, showing the relative locations of Cd (red), Ca (blue), and Zn (green). Reprinted with permission from [59]. Copyright 2023 Elsevier.

SIMS utilizes high-energy primary ion beams to bombard sample surfaces, generating secondary ions for mass spectrometric analysis [56,57,58]. High-resolution secondary ion mass spectrometry (NanoSIMS) was used to investigate the uptake and localization of cadmium sulfide nanoparticles (CdS NPs) in chilis in a comparative foliar and root exposure experiment (Figure 2) [59]. The NanoSIMS images showed that Cd was mainly enriched in the leaf, stem, and root cell walls, providing insights into NP distribution and potential accumulation sites.

### 3.3. Challenges, Method Comparison, and Future Directions

Key challenges in MS-based analysis include differentiating between nanoparticulate and ionic forms of metals, detecting NPs within complex matrices at low concentrations, and preserving NP integrity during analysis. Interference from plant matrix components and the need for specialized instrumentation can also limit the applicability of certain techniques.

spICP-MS provides particle size and number concentration information but requires NPs to be in suspension, limiting its use for solid tissues unless coupled with appropriate extraction methods. LA-ICP-MS enables spatial mapping without extensive sample preparation but may have lower sensitivity for certain elements. SIMS offers high spatial resolution and can analyze solid samples directly but may have limitations in quantification and require complex data interpretation.

Developing methods that can accurately quantify and characterize NPs within intact plant tissues without extensive sample preparation is a significant challenge. Enhancing the sensitivity and specificity of MS techniques, as well as improving data analysis algorithms for complex matrices, will be important. Integration of MS with other analytical methods, such as imaging techniques, could provide more comprehensive insights into NP behavior.

## 4. Microscopy-Based Imaging Techniques

Microscopy offers high spatial resolution for visualizing MNPs within plant tissues, providing insights into their localization and interactions at cellular and subcellular levels.

### 4.1. Electron Microscopy (EM)

**Figure 3:**
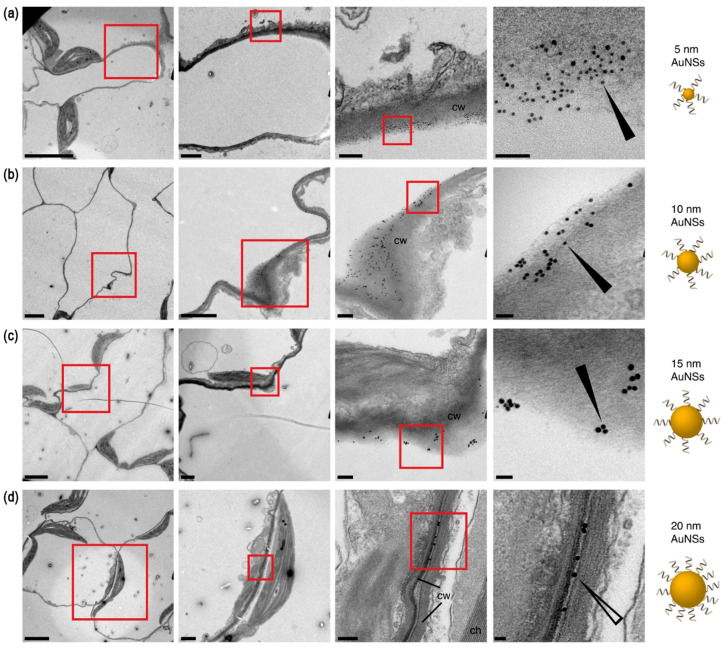
TEM analysis of the subcellular distribution of MNPs in tobacco leaves. Representative TEM images of *N. benthamiana* plants 24 h post-infiltration with DNA-functionalized AuNSs with diameters of 5 nm (**a**), 10 nm (**b**), 15 nm (**c**) and 20 nm (**d**). The images show progressive magnifications from left to right, with the red boxes indicating the magnification areas. Annotations represent the cell wall (cw) and chloroplast (ch). The filled and open arrows indicate NPs associated with a single cell wall or found between cell walls. Scale bars from left to right, 5 µm, 1 µm, 0.2 µm, and 50 nm. Reprinted with permission from [62]. Copyright 2021 Springer Nature.

Transmission electron microscopy (TEM) and scanning electron microscopy (SEM) are used to observe the morphology and localization of MNPs in plant tissues [60,61]. A previous study [62] employed TEM to visualize AuNPs of various sizes and shapes in *Nicotiana benthamiana* leaf cells (Figure 3). These TEM images revealed that rod-shaped AuNPs (AuNRs) could penetrate cell walls and enter cells more effectively than spherical NPs (AuNSs), contributing to understanding the mechanisms of NP uptake. High-resolution SEM was used to observe the internalization of 10 nm AgNPs in green algae [63]. Their images showed NPs localized within cell walls, providing evidence of NP uptake and potential pathways for entry.

### 4.2. Optical Microscopy

Optical microscopy techniques, such as fluorescence microscopy and confocal laser scanning microscopy (CLSM), enable the visualization of fluorescently labeled MNPs in plant tissues [56,62]. A previous study [64] developed fluorescently tagged CeO_2_ NPs by encapsulating the hydrophobic dye DiI within polymer coatings. CLSM visualized the distribution of CeO_2_ NPs in cotton and maize leaves, distinguishing NP fluorescence from plant autofluorescence. A previous study [65] utilized the inherent photoluminescence of Cu_2−x_Se NPs to study their uptake in tomato roots. By observing blue fluorescence under excitation at 405 nm, they confirmed the presence of intact NPs within root tissues.

### 4.3. Two-Photon Fluorescence Microscopy (TPM)

TPM uses longer excitation wavelengths, reducing photodamage and increasing imaging depth compared to conventional fluorescence microscopy. Two-photon fluorescence microscopy was used to observe fresh plant sections exposed to CuO NPs [66,67,68,69]. The detected fluorescence signals attributed to NPs demonstrate the potential of this technique for studying NP uptake in thick plant tissues.

### 4.4. Dark-Field Microscopy (DFM) and Hyperspectral Imaging (HSI)

DFM detects scattered light from NPs, particularly noble MNPs like AuNPs and AgNPs [63,70,71]. HSI provides spectral information for each pixel, aiding in distinguishing NPs based on their optical properties. The combination of DFM with HSI was used to study AgNPs’ interactions with green algae [63]. The strong light scattering from NPs on the cell surface, and hyperspectral analysis allowed for differentiating between NPs and other cellular components.

### 4.5. Challenges, Method Comparison, and Future Directions

Major challenges in microscopy-based techniques include distinguishing NPs from background signals due to plant autofluorescence or other cellular components, potential alterations to samples during preparation (e.g., dehydration in EM), and limitations in imaging depth and resolution. Fluorescent labeling may alter NP properties or introduce artifacts.

Electron microscopy offers high spatial resolution but requires extensive sample preparation and may not be suitable for living tissues. Optical microscopy techniques like CLSM and TPM allow for imaging of living tissues and real-time observation but may suffer from limited resolution and interference from autofluorescence. DFM and HSI provide label-free detection of NPs but may be limited to certain types of NPs with strong scattering properties.

Advancing microscopy techniques to achieve higher resolution and deeper imaging in living plant tissues without introducing artifacts is a significant challenge. Developing non-invasive, label-free imaging methods that can accurately detect and track NPs within complex biological systems will be important. Combining microscopy with other analytical techniques, such as spectroscopy, could enhance the ability to characterize NPs in situ.

## 5. Spectroscopy-Based Analysis

Spectroscopy involves the interaction of electromagnetic radiation with atoms or molecules to obtain structural and compositional information.

### 5.1. X-ray-Based Techniques

X-ray-based techniques are ideal for the in situ detection of MNPs within plant tissues due to their large penetration depths [72,73,74].

#### 5.1.1. Synchrotron Radiation (SR) Techniques

**Figure 4:**
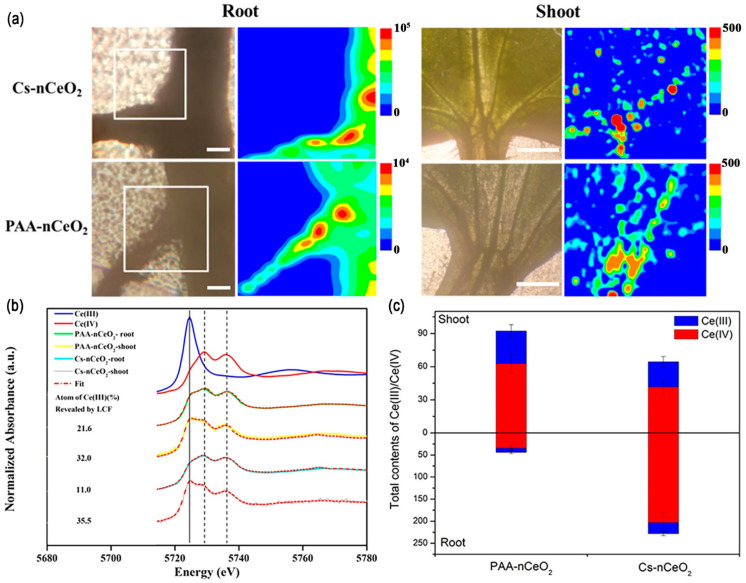
Combining μ-XRF and XANES techniques for analyzing the distribution and chemical speciation of MNPs in cucumber plants. (**a**) μ-XRF images of Ce in cucumber roots and leaves after exposure to 1000 mg/L Cs-nCeO_2_ and PAA-nCeO_2_. The red area of each map corresponds to the maximum concentration of the Ce element. The lateral roots are denoted by the white boxes in the light microscope images. The scale bars for the roots and leaves represent 100 and 500 μm, respectively. Analyses of Ce XANES spectra (**b**) and the total contents of Ce(III) and Ce(IV) in the roots (g/kg) and shoots (mg/kg) (**c**) of cucumber exposed to 1000 mg/L Cs-nCeO_2_ and PAA-nCeO_2_. Reprinted with permission from [78]. Copyright 2019 American Chemical Society.

SR provides intense, tunable X-ray beams for advanced spectroscopic analyses [75,76,77]. The combination of X-ray absorption spectroscopy (XAS) and micro X-ray fluorescence mapping (μ-XRF) was used to study the speciation and distribution of CeO_2_ NPs in cucumber plants (Figure 4) [78]. X-ray absorption near-edge spectroscopy (XANES) distinguished between Ce(III) and Ce(IV) species, indicating the biotransformation of NPs. μ-XRF provided spatial maps showing Ce accumulation in root tips and leaf veins, revealing translocation pathways.

#### 5.1.2. Proton-Induced X-ray Emission (PIXE)

PIXE utilizes proton beams to induce characteristic X-ray emissions from elements within a sample [79,80]. The combination of PIXE with Rutherford backscattering spectrometry (RBS) was used to detect Cu and macronutrients in *Myriophyllum spicatum* exposed to CuO NPs [80]. Elemental maps showed Cu accumulation in parenchyma and vascular tissues, providing insights into uptake and internalization mechanisms.

### 5.2. Infrared and Raman Spectroscopy

Fourier transform infrared (FTIR) spectroscopy and Raman spectroscopy provide information on molecular vibrations, aiding in the analysis of organic components associated with MNPs [81,82,83,84,85,86].

#### 5.2.1. FTIR Spectroscopy

Attenuated total reflectance FTIR (ATR-FTIR) spectroscopy was used to examine the formation of biomolecule coronas on CuO NPs in pumpkin xylem fluid [81]. The spectra indicated the adsorption of organic molecules onto NP surfaces, affecting their stability and transport.

#### 5.2.2. Surface-Enhanced Raman Spectroscopy (SERS)

SERS enhances Raman signals through MNP substrates, enabling sensitive detection of molecular vibrations. An SERS-based method was developed for ultrasensitive quantification and imaging of AgNPs on spinach leaves [87,88]. By attaching Raman reporter molecules to AgNPs, in situ mapping of NP distribution and penetration depth was achieved, demonstrating the technique’s potential for studying NP interactions with plant surfaces.

### 5.3. Laser-Induced Breakdown Spectroscopy (LIBS)

LIBS uses laser ablation to generate plasma emissions from sample surfaces, enabling rapid, multi-element analysis [89]. LIBS was used to study the distribution of CdTe quantum dots (QDs) [90] and photon-upconversion NPs [91] and in duckweed. For an example of CdTe-QDs [90], by mapping the elemental distribution of Cd and Te, the uptake and translocation of QDs within the plant were assessed, highlighting LIBS as a useful tool for the whole-slide imaging of MNPs.

### 5.4. Challenges, Method Comparison, and Future Directions

Spectroscopy-based analysis faces challenges such as limited sensitivity for certain elements, interference from complex plant matrices, and the need for specialized equipment like synchrotron facilities. Distinguishing between different chemical forms of NPs and accurately quantifying them within tissues can be difficult.

X-ray-based techniques offer deep tissue penetration and the ability to determine speciation but require access to synchrotron radiation sources and may have limited spatial resolution. Infrared and Raman spectroscopy provide molecular-level information but can be affected by fluorescence background and may lack sensitivity for low-concentration analytes. SERS enhances sensitivity but depends on the availability of suitable NP substrates and may introduce complexity in sample preparation.

Enhancing the accessibility and sensitivity of spectroscopy techniques for studying MNPs in plants is a key challenge. Developing portable or laboratory-scale instruments that can achieve similar performance to synchrotron-based methods would broaden their applicability. Improving data analysis methods to interpret complex spectra from heterogeneous biological samples will also be important. Integrating spectroscopy with imaging techniques could provide comprehensive insights into NP distribution and transformation. The current techniques for analyzing MNPs in plants and their typical applications are summarized in Table 2. 

## 6. Conclusions and Perspectives

Advancements in analytical methods have significantly improved our ability to study the uptake, translocation, and transformation of MNPs in plants. Effective extraction and pre-processing procedures are critical for preserving NP integrity and ensuring accurate quantification and characterization.

Enzymatic extraction is generally accepted as it removes plant tissues while preserving MNPs. However, limitations related to pH and incomplete digestion necessitate alternative approaches, such as organic solvent-based extraction. Acid digestion remains useful for total metal content analysis but may not preserve NP properties.

MS techniques, particularly ICP-MS and its variants, provide sensitive and specific quantification of MNPs. Spectroscopic methods offer insights into NP speciation, surface properties, and interactions with biomolecules. Microscopy techniques enable high-resolution visualization of MNPs within plant tissues, aiding in understanding their localization and dynamics.

Despite these advances, challenges remain:

Real-Time In Situ Monitoring: Developing techniques that enable simultaneous quantification and localization of MNPs in real time is critical for understanding their behavior. Current methods often require sample preparation that may alter NPs.

Characterization of Biotransformation: During interactions with plants, MNPs may undergo transformations involving biomolecule adsorption or internalization. Few techniques can analyze the organic components associated with transformed NPs with high sensitivity and selectivity.

Future efforts should focus on developing novel analytical tools that combine high sensitivity, selectivity, and spatial resolution without extensive sample preparation. Integrating multiple analytical approaches will enhance our understanding of MNP behavior in plants, informing safe and effective applications of nanotechnology in agriculture and environmental management.

## Figures and Tables

**Table 1:** Extraction methods for investigating MNP uptake by plants.

Matrix	MNP	Extraction Agent	Recovery	Comments	Ref.
Soybean plants	MoS_2_ NPs	HNO_3_, H_2_O_2_	98.1% (Mo total mass)	-	[37]
Rice plants	TiO_2_ NPs	HNO_3_, HCl	-	The size distribution of extracted NP was more similar to the NP control after enzymatic treatment than acid treatment.	[43]
		Macerozyme R-10			
Tomato plants	AuNPs	Macerozyme R-10	79–96% (particle concentration)	The size distribution of extracted particles matched well with the spiked NPs.	[38]
Potato, radish, carrot, and lettuce crops	AuNPs	TMAH	97.2–101.8% (Au mass)	-	[44]
		Macerozyme R-10	<1% (Au mass)		
Lettuce, corn, and kale leaves	AuNPs	Methanol	100% (Au mass)	Based on spICP-MS or ICP-TOF-MS, size distribution, experimental detection limits, and other parameters were evaluated.	[41]
	CuO NPs		80.8–98.6% (Cu mass)		
	ZnO NPs		68.8–94.9% (Zn mass)		
Spanish leaves	AgNPs	4-MBA	-	Following 2 h extraction, the morphology and size of the extracted AgNPs were largely preserved.	[32]

**Table 2:** Techniques for analyzing MNPs in plants.

Technique	Plants	MNP [Original Particle Size]	Sample Preparation	Abundance [LOD/LOQ]	Location [Spatial Revolution]	Morphology, Composition, and Speciation	Ref.
spICP-MS	Radish plants	CeO_2_ NPs [56.9 ± 1.2 nm]	Enzymatic digestion	Particle number concentration	-	Size distribution of NPs	[50]
LA-spICP-MS	Onion cells	AgNPs [60 nm], AuNPs [60 ± 12 nm]	Fresh tissues placed on a glass slide	Particle number concentration	Subcellular location of NPs and distribution of ionic forms [3 μm]	Size distribution of NPs and analysis of NPs and ionic forms	[52]
LA-ICP-MS	*P. glomerata* plants	La_2_O_3_ NPs [15–30 nm]	Dried leaf fixed on a quartz slide	La mass concentration [LOQ: 0.28 μg/g]	La distribution in leaves	Identification of La_2_O_3_ NPs	[53]
HPLC-ICP-MS	Green algae	AuNPs [10, 40 nm]	Ultrasonic disruption	-	-	Speciation and size alteration between Au(III) and AuNPs	[55]
NanoSIMS	Water spinach and pak choi plants	CdS NPs [130 ± 25 × 15 ± 8 nm]	Tissue section	Mass concentration (sq.)	Subcellular distribution of ^+12^C^14^N, ^−32^S, ^40^Ca, and ^+114^Cd [200 nm]	-	[57]
ToF-SIMS	*T. aestivum*, *B. napus*, and *H. vulgare* plants	AgNPs [17 ± 3 nm], CeO_2_ NPs [29 nm]	Frozen tissue section	Isotope concentration (sq.)	3D distribution of Ag and Ce in tissues [LR: 80 nm; DR: 10 nm]	Aggregate size distribution	[58]
TEM	*N. benthamiana* plants	DNA/RNA-AuNRs [13 × 68 nm], AuNSs [5, 10, 15, 20 nm]	Tissue section on copper grids	-	Intracellular location of AuNPs	Shape and size of AuNPs	[62]
AFM	Tomato plants	CS@CH [15–30 nm], CS@OA [15 ± 8 nm]	Fresh tissue fixed on a glass plate	-	-	Adhesion force between NPs and root	[65]
CLSM	Cotton and maize plants	Dil- CeO_2_ NPs [7.5 ± 2.9, 11.7 ± 6.1, 1.8 ± 0.7, 10.8 ± 8.9, 15.6 ± 9.0 nm]	Fixed tissue mounted on slides	-	Colocalization of CeO_2_ NPs and chloroplasts	-	[64]
TPM	Sweet potato roots	CuO NPs [10–100 nm]	Fresh transversal thin sections	-	Tissue location of CuO NPs [600 nm]	-	[66]
DFM-HSI	Green algae	AgNPs [10.0 ± 1.8, 60.8 ± 6.6, 8.8 ± 2.2, 60.8 ± 6.6 nm]	Fresh cell suspension on a slide	-	Subcellular location of NPs	NPs’ identification	[63]
SEM			Tissue section	-	NP location on cell surfaces	Shape and size of NPs	
XANES	Cucumber plants	CeO_2_ NPs [7 nm]	Dried powder	Content of Ce(III) and Ce(IV) (sq.)	-	Transformation between Ce(III) and Ce(IV)	[78]
μ-XRF			Fresh plants	-	Ce distribution on tissue surface [50/100 μm]	-	
EXAFS +XANES	*A. thaliana* (wt. and mut.)	CdS QDs [5 nm]	Dried powder	-	-	Bond of CdS NPs with biomolecules	[75]
PIXE/RBS	*M. spicatum* plants	CuO NPs [64.9 ± 8.5 nm]	Freeze-dried tissue sections	Cu content (sq.)	Tissue distribution of Cu, KCa [2.5 μm]	-	[80]
XPS	Pumpkin xylem fluid	CuO NPs [120 ± 40 × 900 ± 300 nm]	Centrifugation and supernatant deposited on gold-coated silicon	Relative atomic abundances of C_1s_, N_1s_, and O_1s_ [sq.]	-	Elemental composition, corona thickness, and functional groups of CuO NPs	[81]
ATR-FTIR			Suspension	-	-	Dynamic evolution of chemical bonds of corona	
SERS	Spinach leaves	AgNPs [39 ± 4, 50 ± 4, 97 ± 11 nm]	Organic solvent extraction	Mass concentration of AgNPs (sq.)	-	Aggregate of AgNPs	[88]
			Air-dried leaves	-	Penetration depth of AgNPs [10 μm]	Bonds of AgNPs with sulfur-containing biomolecules	
LIBS	Duckweed fronds	Cd-based QDs [4.05.4, 4.0–4.4 nm]	Molded dried leaves glued with epoxide	-	Cd spatial distribution in fronds [200 μm]	-	[90]

LOD: limit of detection, LOQ: limit of quantification, LR: lateral resolution, DR: depth resolution, and sq: semiquantitative. EXAFS: extended X-ray absorption fine structure, XPS: X-ray photoelectron spectroscopy.

## References

[1] Sharma V.K., Filip J., Zboril R., Varma R.S. (2015). Natural inorganic nanoparticles—Formation, fate, and toxicity in the environment. Chem. Soc. Rev..

[2] Nanotechnology Products Database. https://product.statnano.com/.

[3] Wang Q., Astruc D. (2019). State of the art and prospects in metal–organic framework (MOF)-based and MOF-derived nanocatalysis. Chem. Rev..

[4] Borkowska M., Siek M., Kolygina D.V., Sobolev Y.I., Lach S., Kumar S., Cho Y.K., Kandere-Grzybowska K., Grzybowski B.A. (2020). Targeted crystallization of mixed-charge nanoparticles in lysosomes induces selective death of cancer cells. Nat. Nanotechnol..

[5] Zhang P., Jiang Y., Schwab F., Monikh F.A., Grillo R., White J.C., Guo Z., Lynch I. (2024). Strategies for enhancing plant immunity and eesilience Using nanomaterials for sustainable agriculture. Environ. Sci. Technol..

[6] Li R., Chen T., Pan X. (2021). Metal–organic-framework-based materials for antimicrobial applications. ACS Nano.

[7] Kumar A., Choudhary P., Kumar A., Camargo P.H.C., Krishnan V. (2021). Recent advances in plasmonic photocatalysis based on TiO_2_ and noble metal nanoparticles for energy conversion, environmental remediation, and organic synthesis. Small.

[8] Pradas del Real A.E., Castillo-Michel H., Kaegi R., Sinnet B., Magnin V., Findling N., Villanova J., Carrière M., Santaella C., Fernández-Martínez A. (2016). Fate of Ag-NPs in Sewage Sludge after Application on Agricultural Soils. Environ. Sci. Technol..

[9] Gottschalk F., Nowack B. (2011). The release of engineered nanomaterials to the environment. J. Environ. Monit..

[10] Hochella M.F., Spencer M.G., Jones K.L. (2015). Nanotechnology: Nature’s gift or scientists’ brainchild?. Environ. Sci.-Nano.

[11] Kah M., Tufenkji N., White J.C. (2019). Nano-enabled strategies to enhance crop nutrition and protection. Nat. Nanotechnol..

[12] Ma X.M., Geisler-Lee J., Deng Y., Kolmakov A. (2010). Interactions between engineered nanoparticles (ENPs) and plants: Phytotoxicity, uptake and accumulation. Sci. Total Environ..

[13] Avellan A., Yun J., Morais B.P., Clement E.T., Rodrigues S.M., Lowry G.V. (2021). Critical review: Role of inorganic nanoparticle properties on their foliar uptake and in Planta Translocation. Environ. Sci. Technol..

[14] Liu L., Tsyusko O.V., Unrine J.M., Liu S., Liu Y., Guo L., Wei G., Chen C. (2023). Pristine and sulfidized zinc oxide nanoparticles promote the release and decomposition of organic carbon in the legume rhizosphere. Environ. Sci. Technol..

[15] Hu J., Xianyu Y. (2021). When nano meets plants: A review on the interplay between nanoparticles and plants. Nano Today.

[16] Murali M., Gowtham H.G., Singh S.B., Shilpa N., Aiyaz M., Alomary M.N., Alshamrani M., Salawi A., Almoshari Y., Ansari M.A. (2022). Fate, bioaccumulation and toxicity of engineered nanomaterials in plants: Current challenges and future prospects. Sci. Total Environ..

[17] Spielman-Sun E., Avellan A., Bland G.D., Clement E.T., Tappero R.V., Acerbo A.S., Lowry G.V. (2020). Protein coating composition targets nanoparticles to leaf stomata and trichomes. Nanoscale.

[18] Javed R., Khan B., Sharafat U., Bilal M., Galagedara L., Abbey L., Cheema M. (2024). Dynamic interplay of metal and metal oxide nanoparticles with plants: Influencing factors, action mechanisms, and assessment of stimulatory and inhibitory effects. Ecotox. Environ. Safe.

[19] Francis D.V., Abdalla A.K., Mahakham W., Sarmah A.K., Ahmed Z.F.R. (2024). Interaction of plants and metal nanoparticles: Exploring its molecular mechanisms for sustainable agriculture and crop improvement. Environ. Int..

[20] Liu Y.L., Yue L., Wang C.X., Zhu X.S., Wang Z.Y., Xing B.S. (2020). Photosynthetic response mechanisms in typical C3 and C4 plants upon La_2_O_3_ nanoparticle exposure. Environ. Sci.-Nano.

[21] Hong J., Wang L.N., Sun Y.P., Zhao L.J., Niu G.H., Tan W.J., Rico C.M., Peralta-Videa J.R., Gardea-Torresdey J.L. (2016). Foliar applied nanoscale and microscale CeO_2_ and CuO alter cucumber (*Cucumis sativus*) fruit quality. Sci. Total Environ..

[22] Zhang P., Ma Y.H., Zhang Z.Y., He X., Guo Z., Tai R.Z., Ding Y.Y., Zhao Y.L., Chai Z.F. (2012). Comparative toxicity of nanoparticulate/bulk Yb_2_O_3_ and YbCl_3_ to cucumber (*Cucumis sativus*). Environ. Sci. Technol..

[23] Zhu H., Han J., Xiao J.Q., Jin Y. (2008). Uptake, translocation, and accumulation of manufactured iron oxide nanoparticles by pumpkin plants. J. Environ. Monit..

[24] Ahmed B., Rizvi A., Ali K., Lee J., Zaidi A., Khan M.S., Musarrat J. (2021). Nanoparticles in the soil-plant system: A review. Environ. Chem. Lett..

[25] Lv J.T., Christie P., Zhang S.Z. (2019). Uptake, translocation, and transformation of metal-based nanoparticles in plants: Recent advances and methodological challenges. Environ. Sci.-Nano.

[26] Servin A.D., Pagano L., Castillo-Michel H., De la Torre-Roche R., Hawthorne J., Hernandez-Viezcas J.A., Loredo-Portales R., Majumdar S., Gardea-Torresday J., Dhankher O.P. (2017). Weathering in soil increases nanoparticle CuO bioaccumulation within a terrestrial food chain. Nanotoxicology.

[27] Dang F., Huang Y.N., Wang Y.J., Zhou D.M., Xing B.S. (2021). Transfer and toxicity of silver nanoparticles in the food chain. Environ. Sci.-Nano.

[28] Babaei M., Tayemeh M.B., Jo M.S., Yu I., Johari S.A. (2022). Trophic transfer and toxicity of silver nanoparticles along a phytoplankton-zooplankton-fish food chain. Sci. Total Environ..

[29] Liu J.Y., Wang Z.Y., Liu F.D., Kane A.B., Hurt R.H. (2012). Chemical transformations of nanosilver in biological environments. ACS Nano.

[30] Nowack B., Ranville J.F., Diamond S., Gallego-Urrea J.A., Metcalfe C., Rose J., Horne N., Koelmans A.A., Klaine S.J. (2012). Potential scenarios for nanomaterial release and subsequent alteration in the environment. Environ. Toxicol. Chem..

[31] Lv Z.Y., Sun H.D., Du W., Li R.Y., Mao H., Kopittke P.M. (2021). Interaction of different-sized ZnO nanoparticles with maize (*Zea mays*): Accumulation, biotransformation and phytotoxicity. Sci. Total Environ..

[32] Zhang Z.Y., Xia M., Ma C.X., Guo H.Y., Wu W.H., White J.C., Xing B.S., He L.L. (2020). Rapid organic solvent extraction coupled with surface enhanced Raman spectroscopic mapping for ultrasensitive quantification of foliarly applied silver nanoparticles in plant leaves. Environ. Sci.-Nano.

[33] Guo H.Y., Xing B.S., White J.C., Mukherjee A., He L.L. (2016). Ultra-sensitive determination of silver nanoparticles by surface-enhanced Raman spectroscopy (SERS) after hydrophobization-mediated extraction. Analyst.

[34] Sun X.-D., Ma J.-Y., Feng L.-J., Duan J.-L., Yuan X.-Z. (2024). Precise tracking of nanoparticles in plant roots. Nat. Protoc..

[35] Zhang P., Misra S., Guo Z.L., Rehkämper M., Valsami-Jones E. (2019). Stable isotope labeling of metal/metal oxide nanomaterials for environmental and biological tracing. Nat. Protoc..

[36] Zhang P., Guo Z.L., Monikh F.A., Lynch I., Valsami-Jones E., Zhang Z.Y. (2021). Growing Rice (*Oryza sativa*) Aerobically Reduces Phytotoxicity, Uptake, and Transformation of CeO_2_ Nanoparticles. Environ. Sci. Technol..

[37] Li M., Zhang P., Guo Z., Zhao W., Li Y., Yi T., Cao W., Gao L., Tian C.F., Chen Q. (2024). Dynamic transformation of nano-MoS_2_ in a soil–plant system empowers its multifunctionality on soybean growth. Environ. Sci. Technol..

[38] Dan Y.B., Zhang W.L., Xue R.M., Ma X.M., Stephan C., Shi H.L. (2015). Characterization of gold nanoparticle uptake by tomato plants using enzymatic extraction followed by single-particle inductively coupled plasma-mass spectrometry analysis. Environ. Sci. Technol..

[39] Wei W.J., Li L., Gao Y.P., Wang Q., Zhou Y.Y., Liu X., Yang Y. (2021). Enzyme digestion combined with SP-ICP-MS analysis to characterize the bioaccumulation of gold nanoparticles by mustard and lettuce plants. Sci. Total Environ..

[40] Keller A.A., Huang Y.X., Nelson J. (2018). Detection of nanoparticles in edible plant tissues exposed to nano-copper using single-particle ICP-MS. J. Nanopart. Res..

[41] Laughton S., Laycock A., Bland G., von der Kammer F., Hofmann T., Casman E.A., Lowry G.V. (2021). Methanol-based extraction protocol for insoluble and moderately water-soluble nanoparticles in plants to enable characterization by single particle ICP-MS. Anal. Bioanal. Chem..

[42] Laughton S., Laycock A., von der Kammer F., Hofmann T., Casman E.A., Rodrigues S.M., Lowry G.V. (2019). Persistence of copper-based nanoparticle-containing foliar sprays in Lactuca sativa (lettuce) characterized by spICP-MS. J. Nanopart. Res..

[43] Deng Y.Q., Petersen E.J., Challis K.E., Rabb S.A., Holbrook R.D., Ranville J.F., Nelson B.C., Xing B.S. (2017). Multiple method analysis of TiO_2_ nanoparticle uptake in rice (*Oryza sativa* L.) plants. Environ. Sci. Technol..

[44] Malejko J., Godlewska-Zylkiewicz B., Vanek T., Landa P., Nath J., Dror I., Berkowitz B. (2021). Uptake, translocation, weathering and speciation of gold nanoparticles in potato, radish, carrot and lettuce crops. J. Hazard. Mater..

[45] Huang X., Liu H.H., Lu D.W., Lin Y., Liu J.F., Liu Q., Nie Z.X., Jiang G.B. (2021). Mass spectrometry for multi-dimensional characterization of natural and synthetic materials at the nanoscale. Chem. Soc. Rev..

[46] Modlitbová P., Porízka P., Kaiser J. (2020). Laser-induced breakdown spectroscopy as a promising tool in the elemental bioimaging of plant tissues. Trends Anal. Chem..

[47] Laborda F., Gimenez-Ingalaturre A.C., Bolea E., Castillo J.R. (2019). Single particle inductively coupled plasma mass spectrometry as screening tool for detection of particles. Spectroc. Acta Pt. B-Atom. Spectr..

[48] Laborda F., Bolea E., Jiménez-Lamana J. (2016). Single particle inductively coupled plasma mass spectrometry for the analysis of inorganic engineered nanoparticles in environmental samples. Trends Environ. Anal. Chem..

[49] Wu J., Sun J., Bosker T., Vijver M.G., Peijnenburg W. (2023). Toxicokinetics and particle number-based trophic transfer of a metallic nanoparticle mixture in a terrestrial food chain. Environ. Sci. Technol..

[50] Wojcieszek J., Jiménez-Lamana J., Bierla K., Ruzik L., Asztemborska M., Jarosz M., Szpunar J. (2019). Uptake, translocation, size characterization and localization of cerium oxide nanoparticles in radish (*Raphanus sativus* L.). Sci. Total Environ..

[51] Gao X., Kundu A., Persson D.P., Szameitat A., Minutello F., Husted S., Ghoshal S. (2023). Application of ZnO nanoparticles encapsulated in mesoporous silica on the abaxial side of a *Solanum lycopersicum* leaf enhances Zn uptake and translocation via the phloem. Environ. Sci. Technol..

[52] Yamashita S., Yoshikuni Y., Obayashi H., Suzuki T., Green D., Hirata T. (2019). Simultaneous determination of size and position of silver and gold nanoparticles in onion cells using laser ablation-ICP-MS. Anal. Chem..

[53] Neves V.M., Heidrich G.M., Rodrigues E.S., Enders M.S.P., Muller E.I., Nicoloso F.T., de Carvalho H.W.P., Dressler V.L. (2019). La_2_O_3_ nanoparticles: Study of uptake and distribution in *Pfaffia glomerata* (Spreng.) pedersen by LA-ICP-MS and *μ*-XRF. Environ. Sci. Technol..

[54] Li Z., Yan W., Li Y., Xiao Y., Shi Y., Zhang X., Lei J., Min K., Pan Y., Chen X. (2023). Particle size determines the phytotoxicity of ZnO nanoparticles in rice (*Oryza sativa* L.) revealed by spatial imaging techniques. Environ. Sci. Technol..

[55] Malejko J., Swierzewska N., Bajguz A., Godlewska-Zylkiewicz B. (2018). Method development for speciation analysis of nanoparticle and ionic forms of gold in biological samples by high performance liquid chromatography hyphenated to inductively coupled plasma mass spectrometry. Spectroc. Acta Pt. B-Atom. Spectr..

[56] Zhao B., Luo Z.X., Zhang H.L., Zhang H. (2022). Imaging tools for plant nanobiotechnology. Front. Genome Ed..

[57] Ouyang X.X., Ma J., Zhang R., Li P., Gao M., Sun C.Q., Weng L.P., Chen Y.L., Yan S., Li Y.T. (2022). Uptake of atmospherically deposited cadmium by leaves of vegetables: Subcellular localization by NanoSIMS and potential risks. J. Hazard. Mater..

[58] Wagener S., Jungnickel H., Dommershausen N., Fischer T., Laux P., Luch A. (2019). Determination of nanoparticle uptake, distribution, and characterization in plant root tissue after realistic long-term exposure to sewage sludge using information from mass spectrometry. Environ. Sci. Technol..

[59] Ouyang X.X., Ma J., Liu Y., Li P., Wei R.F., Chen Q.S., Weng L.P., Chen Y.L., Li Y.T. (2023). Foliar cadmium uptake, transfer, and redistribution in Chili: A comparison of foliar and root uptake, metabolomic, and contribution. J. Hazard. Mater..

[60] Zhang P., Ma Y.H., Xie C.J., Guo Z.L., He X., Valsami-Jones E., Lynch I., Luo W.H., Zheng L.R., Zhang Z.Y. (2019). Plant species-dependent transformation and translocation of ceria nanoparticles. Environ. Sci.-Nano.

[61] Wang H., Zhao Y., Yin S., Dai Y., Zhao J., Wang Z., Xing B. (2023). Antagonism toxicity of CuO nanoparticles and mild ocean acidification to marine algae. J. Hazard. Mater..

[62] Zhang H., Goh N.S., Wang J.W., Pinals R.L., González-Grandío E., Demirer G.S., Butrus S., Fakra S.C., Flores A.D., Zhai R. (2022). Nanoparticle cellular internalization is not required for RNA delivery to mature plant leaves. Nat. Nanotechnol..

[63] Sekine R., Moore K.L., Matzke M., Vallotton P., Jiang H.B., Hughes G.M., Kirby J.K., Donner E., Grovenor C.R.M., Svendsen C. (2017). Complementary imaging of silver nanoparticle interactions with green algae: Dark-field microscopy, electron microscopy, and nanoscale secondary ion mass spectrometry. ACS Nano.

[64] Hu P.G., An J., Faulkner M.M., Wu H.H., Li Z.H., Tian X.L., Giraldo J.P. (2020). Nanoparticle charge and size control foliar delivery efficiency to plant cells and organelles. ACS Nano.

[65] Sharma S., Muddassir M., Muthusamy S., Vaishnav P.K., Singh M., Sharma D., Kanagarajan S., Shanmugam V. (2020). A non-classical route of efficient plant uptake verified with fluorescent nanoparticles and root adhesion forces investigated using AFM. Sci. Rep..

[66] Bonilla-Bird N.J., Paez A., Reyes A., Hernandez-Viezcas J.A., Li C., Peralta-Videa J.R., Gardea-Torresdey J.L. (2018). Two-photon microscopy and spectroscopy studies to determine the mechanism of copper oxide nanoparticle uptake by sweetpotato roots during postharvest treatment. Environ. Sci. Technol..

[67] Wang Y., Deng C., Cota-Ruiz K., Peralta-Videa J.R., Sun Y., Rawat S., Tan W., Reyes A., Hernandez-Viezcas J.A., Niu G. (2020). Improvement of nutrient elements and allicin content in green onion (*Allium fistulosum*) plants exposed to CuO nanoparticles. Sci. Total Environ..

[68] Deng C., Wang Y., Cota-Ruiz K., Reyes A., Sun Y., Peralta-Videa J., Hernandez-Viezcas J.A., Turley R.S., Niu G., Li C. (2020). Bok choy (*Brassica rapa*) grown in copper oxide nanoparticles-amended soils exhibits toxicity in a phenotype-dependent manner: Translocation, biodistribution and nutritional disturbance. J. Hazard. Mater..

[69] Deng C., Wang Y., Navarro G., Sun Y., Cota-Ruiz K., Hernandez-Viezcas J.A., Niu G., Li C., White J.C., Gardea-Torresdey J. (2022). Copper oxide (CuO) nanoparticles affect yield, nutritional quality, and auxin associated gene expression in weedy and cultivated rice (*Oryza sativa* L.) grains. Sci. Total Environ..

[70] Xie X.D., Hu X.J., Li Q., Yin M., Song H.Y., Hu J., Wang L.H., Fan C.H., Chen N. (2020). Unraveling cell-type-specific targeted delivery of membrane-camouflaged nanoparticles with plasmonic imaging. Nano Lett..

[71] Ye Y., Reyes A.M., Li C., White J.C., Gardea-Torresdey J.L. (2023). Mechanistic insight into the internalization, distribution, and autophagy process of manganese nanoparticles in *Capsicum annuum* L.: Evidence from orthogonal microscopic analysis. Environ. Sci. Technol..

[72] Zhu S., Wang Y.L., Chen C.Y. (2022). In situ analysis of the fate and behavior of inorganic nanomaterials in biological systems by synchrotron radiation X-ray probe techniques. Curr. Anal. Chem..

[73] Vijayan P., Willick I.R., Lahlali R., Karunakaran C., Tanino K.K. (2015). Synchrotron radiation sheds fresh light on plant research: The use of powerful techniques to probe structure and composition of plants. Plant Cell Physiol..

[74] Castillo-Michel H.A., Larue C., Pradas del Real A.E., Cotte M., Sarret G. (2017). Practical review on the use of synchrotron based micro- and nano- X-ray fluorescence mapping and X-ray absorption spectroscopy to investigate the interactions between plants and engineered nanomaterials. Plant Physiol. Biochem..

[75] Marmiroli M., Lepore G.O., Pagano L., d’Acapito F., Gianoncelli A., Villani M., Lazzarini L., White J.C., Marmiroli N. (2020). The fate of CdS quantum dots in plants as revealed by extended X-ray absorption fine structure (EXAFS) analysis. Environ. Sci.-Nano.

[76] Rodrigues S., Avellan A., Bland G.D., Miranda M.C.R., Larue C., Wagner M., Moreno-Bayona D.A., Castillo-Michel H., Lowry G.V., Rodrigues S.M. (2024). Effect of a zinc phosphate shell on the uptake and translocation of foliarly applied ZnO nanoparticles in pepper plants (*Capsicum annuum*). Environ. Sci. Technol..

[77] Freitas M.N., Guerra M.B.B., Adame A., Moraes T.F., Junior J.L., Pérez C.A., Abdala D.B., Cicero S.M. (2020). A first glance at the micro-ZnO coating of maize (*Zea mays* L.) seeds: A study of the elemental spatial distribution and Zn speciation analysis. J. Anal. At. Spectrom..

[78] Liu M.Y., Feng S., Ma Y.H., Xie C.J., He X., Ding Y.Y., Zhang J.Z., Luo W.H., Zheng L.R., Chen D.L. (2019). Influence of surface charge on the phytotoxicity, transformation, and translocation of CeO_2_ nanoparticles in cucumber plants. ACS Appl. Mater. Interfaces.

[79] Larue C., Castillo-Michel H., Stein R.J., Fayard B., Pouyet E., Villanova J., Magnin V., Pradas del Real A.E., Trcera N., Legros S. (2016). Innovative combination of spectroscopic techniques to reveal nanoparticle fate in a crop plant. Spectroc. Acta Pt. B-Atom. Spectr..

[80] Dumont E.R., Elger A., Azéma C., Michel H.C., Surble S., Larue C. (2022). Cutting-edge spectroscopy techniques highlight toxicity mechanisms of copper oxide nanoparticles in the aquatic plant *Myriophyllum spicatum*. Sci. Total Environ..

[81] Borgatta J.R., Lochbaum C.A., Elmer W.H., White J.C., Pedersen J.A., Hamers R.J. (2021). Biomolecular corona formation on CuO nanoparticles in plant xylem fluid. Environ. Sci.-Nano.

[82] Candan F., Markushin Y., Ozbay G. (2022). Uptake and presence evaluation of nanoparticles in *Cicer arietinum* L. by infrared spectroscopy and machine learning techniques. Plants.

[83] Ntziachristos V. (2010). Going deeper than microscopy: The optical imaging frontier in biology. Nat. Methods.

[84] Guo H.Y., He L.L., Xing B.S. (2017). Applications of surface-enhanced Raman spectroscopy in the analysis of nanoparticles in the environment. Environ. Sci.-Nano.

[85] Savassa S.M., Castillo-Michel H., Pradas del Real A.E., Reyes-Herrera J., Marques J.P.R., de Carvalho H.W.P. (2021). Ag nanoparticles enhancing Phaseolus vulgaris seedling development: Understanding nanoparticle migration and chemical transformation across the seed coat. Environ. Sci.-Nano.

[86] Cupil-Garcia V., Li J.Q., Norton S.J., Odion R.A., Strobbia P., Menozzi L., Ma C., Hu J., Zentella R., Boyanov M.I. (2023). Plasmonic nanorod probes’ journey inside plant cells for in vivo SERS sensing and multimodal imaging. Nanoscale.

[87] Guo H.Y., Zhang Z.Y., Xing B.S., Mukherjee A., Musante C., White J.C., He L.L. (2015). Analysis of silver nanoparticles in antimicrobial products using surface-enhanced raman spectroscopy (SERS). Environ. Sci. Technol..

[88] Zhang Z.Y., Shang H.P., Xing B.S., He L.L. (2021). In situ and real time investigation of foliarly applied silver nanoparticles on and in spinach leaves by surface enhanced Raman spectroscopic mapping. Anal. Methods.

[89] Galbács G. (2015). A critical review of recent progress in analytical laser-induced breakdown spectroscopy. Anal. Bioanal. Chem..

[90] Modlitbová P., Novotny K., Porízka P., Klus J., Lubal P., Zlámalová-Gargosová H., Kaiser J. (2018). Comparative investigation of toxicity and bioaccumulation of Cd-based quantum dots and Cd salt in freshwater plant *Lemna minor* L.. Ecotox. Environ. Safe.

[91] Modlitbová P., Hlavácek A., Svestková T., Porízka P., Simoníkova L., Novotny K., Kaiser J. (2019). The effects of photon-upconversion nanoparticles on the growth of radish and duckweed: Bioaccumulation, imaging, and spectroscopic studies. Chemosphere.

